# Survivors Overcoming and Achieving Resiliency (SOAR): Mindful Eating Practice for Breast Cancer Survivors in a Virtual Teaching Kitchen

**DOI:** 10.3390/nu15194205

**Published:** 2023-09-29

**Authors:** Sherri Huang, Diane Riccardi, Sonya Pflanzer, Laura S. Redwine, Heewon L. Gray, Tiffany L. Carson, Marc McDowell, Zachary Thompson, Jesse J. Hubbard, Smitha Pabbathi

**Affiliations:** 1 Morsani College of Medicine, University of South Florida, Tampa, FL 33620, USA; 2 H. Lee Moffitt Cancer Center & Research Institute, Tampa, FL 33612, USA; 3 Osher Center for Integrative Health, Department of Family Medicine and Community Health, Miller School of Medicine, University of Miami, Miami, FL 33134, USA; 4 College of Public Health, University of South Florida, Tampa, FL 33612, USA; 5 Department of Health Outcomes and Behavior, Division of Population Sciences, Moffitt Cancer Center, Tampa, FL 33612, USA

**Keywords:** cancer survivorship, breast cancer, teaching kitchens, mindful eating, mindfulness, integrative medicine, plant-based diets, multidisciplinary care, food attitude, dietary behavior

## Abstract

The practice of mindful eating brings awareness to food choices, brings attention to the eating experience, and encourages selecting and preparing food that is both satisfying and nourishing. We examined mindful eating in breast cancer survivors following a 9-week, multidisciplinary virtual teaching kitchen intervention called Survivors Overcoming and Achieving Resiliency (SOAR). SOAR engaged participants through weekly cooking classes that also taught multiple domains of mindfulness. Participants (*n* = 102) were breast cancer survivors and completed the Mindful Eating Questionnaire (MEQ) prior to and after completion of the intervention. Linear regression analyses examined relationships between the aspects of mindful eating and body mass index (BMI). Wilcoxon (paired) rank sum tests evaluated the significance of the change in the MEQ total sum and subscales scores. A total of 102 participants completed both the pre- and post-intervention surveys. The mean change between the pre- and post-SOAR MEQ summary scores was 0.12 (sd = 0.30; Wilcoxon *p*-value = 0.0003). All MEQ subscale scores significantly increased with the exception of the distraction subscale. The MEQ summary scores increased for participants across both BMI stratifications. The SOAR teaching kitchen represents one of the first interventions that is tailored for breast cancer survivors and combines behavioral strategies from mindful eating training to nutritional knowledge and culinary medicine pedagogy in a virtual teaching kitchen. Further research is needed to examine whether mindful eating practices among cancer survivors result in sustainable healthy eating behaviors and food choices consistent with the cancer risk reduction guidelines.

## 1. Introduction

With the development of novel cancer treatments and improvement in existing therapies, overall cancer survival has increased in the past decade, and the number of cancer survivors is estimated to increase by 24.4% in another decade [1]. As such, the care of cancer survivors has become an increasingly important field focusing on surveillance for cancer recurrence, screening for second malignancies, and monitoring long-term and late treatment effects to improve survival outcomes and the quality of life. Therefore, one critical but challenging aspect of cancer survivorship care for practitioners is promoting the modification of behavioral risk factors for cancer prevention, such as by providing guidance on behaviors towards healthy eating behaviors. 

Higher diet quality and physical exercise are associated with reduced mortality and improved quality of life [2,3]. The most effective interventions for improving the quality of life in breast cancer survivors combine healthy dietary choices and physical activity [4,5,6]. Dietary modifications alone have benefits as well. Diet quality was inversely related to all-cause mortality in a prospective study of breast cancer survivors [7]. Diet alone improved scores for subjective vitality (a measure of energy and fatigue) in a study that evaluated the role of diet and exercise alone and in combination on the quality of life in overweight and obese breast cancer survivors [4]. There may also be improved cognitive benefits from following healthy diets [8]. 

Specific diets, such as plant-based and Mediterranean diets, are associated with a better physical, mental, emotional, and social quality of life in breast cancer survivors [7]. One randomized clinical trial showed the benefits of a diet rich in whole grains, fruits, vegetables, and omega-3 fatty acid-rich foods in decreasing fatigue and improving sleep quality for breast cancer survivors [9]. Fruits and vegetables contain an abundance of a variety of compounds from phenolic acids, flavonoids, lignans, and carotenoids to stilbenes that have been shown to reduce the risk of breast cancer progression and recurrence. Indeed, numerous studies have shown the effect of plant-based diets on breast cancer prevention and recurrence. For example, in one cross-sectional study, 3646 women were administered food frequency surveys and followed longitudinally over a median of 9.5 years after breast cancer diagnosis; in this study, healthful diets were as defined by a higher frequency of plant-based foods (i.e., whole grains, fruits, and vegetables) over unhealthful foods (i.e., fruit juices, refined grains, and sweets) and was associated with reduced all-cause mortality [10]. Plasma levels of carotenoid concentrations were inversely related to the risk of new breast cancer events in women previously treated for breast cancer [11]. In the Women’s Healthy Eating and Living (WHEL) study, participants with high carotenoid intake demonstrated the lowest rate of overall events which included local/regional recurrence, distant recurrent, and new primary breast cancer compared to participants with low carotenoid intake [12]. Finally, a deficiency in beta carotene is correlated with the incidence of triple-negative breast cancer [13].

At the cellular level, flavonoids and polyphenols in plants can regulate tumor suppressor genes through epigenetic modifications. For example, polyphenols and flavonoids exert multiple cellular effects that prevent the development or progression of cancer, including antioxidant actions, epigenetic modifications that activate pathways of DNA repair, and inhibition of cellular proliferation [14,15]. Given the many benefits of the plant-based diet, the National Comprehensive Cancer Network (NCCN) and the American Society for Clinical Oncology (ASCO) recommend plant-based over animal-based diets. Similarly, the American Cancer Society (ACS) recommends diets heavy in plant foods and limiting or avoiding processed and/or red meats, refined grain products, and sugar-sweetened drinks. The American Institute for Cancer Research (AICR) echoes these recommendations and adds support for a whole-food, plant-based diet as beneficial for cancer prognosis [16]. To combat the global rise in obesity and diabetes, the WHO recommends for adults a healthy diet comprising whole grains, nuts, legumes, vegetables, and fruits, with at least five servings of fruits and vegetables daily. It also advocates for a low-sugar, low-salt, and low-fat diet [17]. 

In addition, plant-based diets help maintain and promote weight loss, which also has benefits for cancer prevention. Obesity correlates with increased body adipose, which consequently upregulates a pro-inflammatory state mediated by the altered adipokines profile and the production of pro-inflammatory cytokines [18]. The development of metabolic syndrome also plays a role, such as via the increased production of insulin, which promotes cellular proliferation through binding of insulin receptor; and increased production of the insulin growth factor, which activates pathways of proliferation, angiogenesis, and anti-apoptosis [19]. Finally, increased adiposity is correlated with widespread epigenetic changes and genome-wide methylation changes, including at the promoters of tumor suppressors [20]. Causal analysis suggests that adiposity causes these methylome changes, rather than vice versa [20]. Together, these studies emphasize the phenotypic benefits of plant-based diets and having a normal BMI which have their basis from multiple cellular effects. Moreover, cancer survivors experience weight gain during cancer treatment, and therefore, plant-based diets may represent a way to counter weight gain and the increased adiposity eventually leading to the pro-inflammatory state that promotes cancer recurrence [21].

However, many cancer survivors face environmental and social barriers that impact their ability to adhere to the dietary recommendations set forth by the leading cancer agencies, and post-treatment side effects such as changes in gustatory taste, bowel changes, and fatigue may influence motivation. Moreover, cancer survivors look to healthcare providers and peers to guide and support them in following lifestyle changes [22,23,24]. Nutritional support programs may therefore motivate and increase the understanding of strategies to improve eating behaviors in cancer survivors. Growing research suggests that health education interventions incorporating integrative health components, including mindfulness-based strategies, meditation, and yoga, can enhance receptiveness and adherence to behavior changes such as diet and physical activity and reduce harmful substance use [25,26,27,28]. 

Mindfulness-based strategies target the experience of eating, bringing mindfulness to food choices and the eating process. According to The Center for Mindful Eating (TCME), the practice of mindful eating promotes awareness of our thoughts, feelings, and physical sensations related to eating, reconnecting us with our innate inner wisdom about hunger and satiety [29]. Because mindful eating aims to increase intentional food choices and reduce emotional eating, integrating mindful eating approaches may help prevent weight gain by developing an awareness of the sensory properties of foods and internal indicators of hunger and fullness [30]. Although the effect of mindful eating as a weight-loss strategy has not been well-studied in cancer survivorship, pilot studies show promise that a slower, more intentional way of eating could help impact unhealthy weight in breast cancer survivors [31,32]. By impacting weight loss, mindful eating may exert longstanding cellular effects, as discussed above. 

The educational use of teaching kitchens has been successful in improving health outcomes such as HbA1c levels, blood pressure, and lipid levels in non-cancer patients [33]. The essence of a teaching kitchen, whether existing in the physical or virtual space, is best described by The Teaching Kitchen Collaborative as a learning laboratory for life skills where we can teach participants to eat, cook, move, and think more healthfully [34,35]. This pairs nutrition education with hands-on culinary skills, incorporates tenets of physical exercise along with mindfulness, and highlights behavior change principles [36]. In light of the promising results of both integrative health modalities and education with teaching kitchens, we sought to promote healthy lifestyles in breast cancer survivors at our cancer center by creating Survivors Overcoming and Achieving Resiliency (SOAR), a nine-week virtual teaching kitchen incorporating nutrition education, meditation, exercise, yoga, mindfulness, and art into weekly cooking classes. Given the consensus on the benefits of a plant-based diet which has molecular effects on cancer progression and recurrence, SOAR focuses on plant-based and/or Mediterranean recipes to stay consistent with the nutrition guidelines for cancer survivors and prevention. In this study, we examined the role of SOAR in promoting mindful eating as a potential method of encouraging a healthy lifestyle for breast cancer survivors.

## 2. Materials and Methods

### 2.1. Participants

Participants were included in the study if they were female, 18 years or older, spoke English, and had a history of stage 0, I, II, or III breast cancer with at least six months since their most recent cancer treatment. Participants were excluded if they were currently undergoing active treatment such as surgery, chemotherapy, and/or radiation. Participants were recruited between fall 2020 and spring 2023 through Moffitt Cancer Center’s breast cancer and survivorship clinics, social media, websites, and flyers posted in the hospital. During this recruitment period, six cohorts with 102 participants completed the program. 

### 2.2. Procedures

Potential participants underwent a preliminary phone screening, and eligibility was confirmed at an in-person visit. Eligible participants completed the informed consent process and were enrolled into the study. Participants completed a demographics survey and self-report measures including the Mindful Eating Questionnaire (MEQ) and Food Attitudes and Behaviors (FAB) survey prior to and after completion of the 9-week intervention [37,38]. Participants’ height and weight were self-reported, and body mass index (BMI) was calculated. Informed consent and study procedures were conducted in accordance with the ethical standards of the Moffitt Cancer Center and standards set forth by the Declaration of Helsinki. Participants received compensation for study participation in the form of grocery and Amazon gift-cards. 

### 2.3. SOAR Intervention

#### 2.3.1. Overview

The SOAR virtual teaching kitchen was created for breast cancer survivors by a team of experts employed at Moffitt Cancer Center. All had extensive experience and expertise in caring for cancer patients and survivors. 

#### 2.3.2. Professional Team

The team included a licensed registered dietitian, a certified culinary medicine specialist (CCMS) and physician assistant, a licensed clinical social worker (LCSW) experienced in mindfulness techniques, a physical and occupational therapist, a certified yoga therapist (C-IAYT), an artist in residence, and a physician board certified in obesity medicine and internal medicine. The team was overseen by the Medical Director for the Cancer Survivorship Clinic. Specific roles of this multidisciplinary team included: (1) Physical and occupational therapist: Discussed the many benefits of exercise after cancer treatment and helped participants design an exercise plan tailored to the fitness and energy level of the patient. (2) LCSW: Trained in a residential Mindfulness-Based Stress Reduction (MBSR) program, the social worker provided mindfulness classes that addressed mindfulness moments in the activities of shopping for, preparing, and consuming a nourishing, healthful meal. In addition, he collaborated with the artist in residence to develop and facilitate a mindfulness meditation to add to the process of creating mindful art in the last session. (3) Yoga therapist: The Patient Wellness Coordinator and certified yoga therapist in Moffitt’s Integrative Medicine Program led the class in a yoga exercise intentionally using the breath to help the body move and stretch. Time at the end of the practice was devoted to reflection. Tailored to the cancer patient, these mind–body therapies were made simple and accessible so that patients could practice the exercise at any point in their cancer journey. (4) Artist in Residence: Led by an artist with Moffitt’s Arts in Medicine Program, the final session introduced expressive art as a personal healing and meditative practice. 

#### 2.3.3. Structure and Topics

SOAR was created over a 2-year pilot period and evolved into a specific 9-week breast cancer survivorship curriculum to educate and support mindful eating and other healthy habits. The 9-week intervention comprises weekly topics including mindful eating, nutrition, emotional health and well-being, exercise, cancer survivorship, meditation, and moving forward after cancer treatment. 

Each session identified a learning objective for the participants. Experiential exercises were adapted to address cancer survivorship. Mindfulness exercises included meditation, mindful movement, and a mindful eating exercise. SOAR sessions were administered by a registered dietitian and culinary medicine specialist biannually in the spring and fall. 

All recipes were from AICR.org and promote a diet that is categorized as plant-based, whole foods, and Mediterranean. The recipes incorporated fruits (lime, strawberry, pomegranate, mango, avocado), whole grains (overnight oats), legumes (lentils), vegetables (spinach, mushrooms, salad), and fresh herbs. The specific session agenda including the session topic, activities, and healthy recipes are shown in Supplementary Table S1.

### 2.4. Outcome Measures

#### Mindful Eating Questionnaire (MEQ) [37]

The five subscales include disinhibition, awareness, external cues, emotional response, and distraction. Items are rated on a scale from 1 (never/rarely) to 4 (usually/always) and are averaged for a total score, with a higher score signifying more mindful eating. The subscales have good internal consistency reliability, with Cronbach’s alpha ranging from 0.64 to 0.83. The reliability of the MEQ summary score (i.e., mean of the five subscale scores) was good (0.64). The correlations between the subscales and summary score ranged from 0.57 to 0.71.

BMI for each participant was calculated from the self-reported height and weight of the participants (weight (lb)/[height (in)]^2^ × 703). BMI was categorized into non-obese (less than 30.0) and obesity (≥30.0).

### 2.5. Statistical Analyses

Patient characteristics were summarized using descriptive statistics including median and range for continuous measures and proportions and frequencies for categorical measures. Summary statistics were calculated for the total and subscales at the pre- and post-survey timepoints. Summary statistics were calculated for the change between the pre- and post-SOAR surveys (post–pre-scores).

Wilcoxon (paired) rank sum tests were used to assess the significance of the change in the total sum and subscales of the MEQ between the pre- and post-surveys without adjusting for BMI or interactions. Wilcoxon tests were also performed, stratifying by BMI on the summary score and subscales. Linear mixed effects models were also run for the summary score and for each subscale score with random intercepts to address the variation between subjects, adjusting for BMI and interaction with the program effect over time. 

Graphical methods include boxplots for the summary score and subscales. 

## 3. Results

A total of 102 participants completed both the pre- and post-intervention surveys between fall of 2020 and spring of 2023. A total of 80% percent of participants attended six or more of the nine sessions. As shown in Table 1, fifty-one (50%) had a BMI of less than 30. Most reported their gender as female with two reporting ‘other’. All participants were 35 or older, with 68% being 55 or older. The majority of the participants were White (81%), while 13% reported their race as Black, and 7% reported their ethnicity as being Hispanic. Most had some college education (26%) or a college degree (65%). 

**Figure 1:**
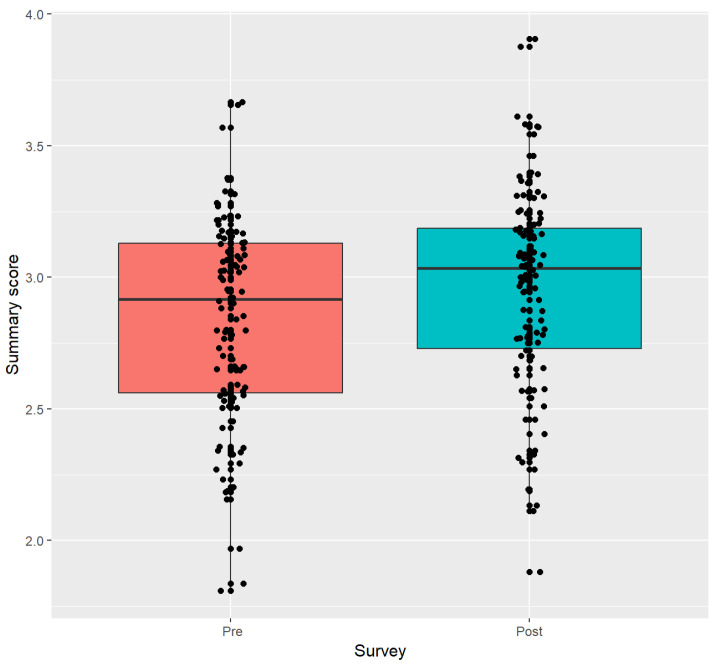
Boxplots of MEQ summary scores.

As shown in Table 2, the mean change in the summary score (post–pre) was 0.12 with a standard deviation (sd) of 0.30 (Wilcoxon *p*-value = 0.0003). All subscales had a significant increase in change with the exception of the distraction subscale (Table 2). The mean change for the awareness subscale was 0.15 (*p*-value = 0.0023). The disinhibition change was 0.12 (*p*-value = 0.004). The emotional change from the baseline was 0.17 (*p*-value = 0.0034), and the external subscale had an increase of 0.15 (*p*-value = 0.0022). The boxplots in Figure 1 show the distribution of the pre- and post-intervention survey scores. 

The linear mixed-effects model found that after adjusting for BMI and the interaction of BMI and time, the summary score increased significantly from baseline to post-intervention by 0.118 with a *p*-value of 0.0069. There was not a significant change in the subscale scores for awareness and distraction. The disinhibition subscale score increased by 0.185 (*p*-value = 0.0016), and the emotional subscale score increased by 0.243 (*p*-value = 0.0027). For the external subscale, the change in the score was 0.162 (*p*-value = 0.0237), and the BMI term was not different from zero. 

The Wilcoxon rank sum tests used to examine changes in the summary score and subscales, stratifying by BMI, found that the participants with a BMI < 30 had an increase in the MEQ summary score, awareness, and external subscales. Those with a BMI greater than or equal to 30 had increases in the summary score, disinhibition, emotional, and external subscales. 

## 4. Discussion

Cancer survivors face several obstacles to maintaining the healthy diet and exercise recommendations set forth by leading cancer agencies. These obstacles include environmental and social barriers along with physical changes post-treatment such as gustatory changes and fatigue. Previous interventions to promote healthy diets and physical activity in cancer survivors largely focused on recommendations for Mediterranean, low-carbohydrate, and/or low-cholesterol diets and structured exercise programs or a combination of dietary and exercise interventions [5]. A few programs combined pharmacologic therapies to assist with weight loss. Existing lifestyle intervention programs for cancer survivors have also evaluated the role of mindfulness on outcomes such as depression, anxiety, and stress [31,39,40]. As of this writing, extant mindful eating programs include Thomas et al.’s 2019 study which evaluated the Mindfulness-Oriented Recovery Enhancement (MORE) program on targeting food attention biases in overweight or obese cancer survivors [41]. Another study, Sattler et al. 2018, evaluated mindful eating in obese breast cancer survivors [29]. Chung et al. 2016 evaluated a mindful eating program specifically in breast cancer survivors who identified as African American [28]. Therefore, despite strong support for the potential for mindful eating to promote healthier eating habits, limited studies to date have evaluated the effect of a mindful eating intervention on the eating experience of cancer survivors. 

To address the need to promote healthy eating habits in breast cancer survivors at our cancer center, we created a virtual teaching kitchen, SOAR. SOAR focuses on teaching a plant-based and Mediterranean diet; this premise employs concepts based on knowledge about nutrigenomics and genetic and epigenetic pathways that underlie cancer recurrence and progression. As previously discussed, the phytochemicals in plant-based diets have been demonstrated to modulate epigenetic pathways that regulate cancer development, progression, and recurrence [14,15]. By promoting mindful eating, SOAR aims to encourage mindful dietary planning which may also have sustainable effects on unhealthy weight and, therefore, may have cellular changes with respect to the production of pro-inflammatory cytokines.

Mindful eating has not been extensively evaluated for its impact on healthy diet guidelines adherence, and this study represents one of the first of its kind that is tailored for breast cancer survivors and combines behavioral strategies including mindful eating training, nutritional knowledge, and culinary medicine pedagogy in a virtual teaching kitchen. A team of experts in culinary medicine, dietetics, internal medicine, physical and occupational therapy, yoga, and social work as well as an artist in residence specialized in different domains of physical and mental well-being and delivered a mindful eating intervention that was measured with the MEQ. Although we were not able to correlate different activities of mindfulness to different domains of the MEQ (disinhibition, awareness, emotional, external cues, and distraction), we found that the combination and multidisciplinary strategy of SOAR was efficacious in critical aspects of mindfulness, in particular, awareness and intention to food choices, while reducing emotional and external eating. Consistent with this, the summary scores of the MEQ showed that the participants overall demonstrated increases in the average scores in all domains, with the exception of the distraction domain. Further qualitative studies such as with focus groups may reveal the reason for stagnancy with the distraction domain. 

Besides the multidisciplinary approach, another aspect of SOAR is its accessibility via the virtual kitchen and robust participant engagement. The virtual kitchen taught survivors and, by extension, their families to prepare plant-forward meals in the comfort of their own homes. Participants were taught culinary techniques and invited to cook along in 90-minute segments in two of the nine weeks with the culinary medicine specialist and the dietitian to make a plant-based meal. The virtual kitchen also allowed a space in which the participants could feasibly engage with different experts in the ongoing discussion and in a group-based setting. Participants were also encouraged to send the team photos of the plant-based SOAR meals they cooked during the week, and photos were compiled into a slideshow to share with other participants in the next week. Finally, the physical and occupational therapist provided customized exercise plans for the participants. 

Together, the accessibility and interactivity of SOAR likely address the environmental and social barriers, obstacles discussed in the literature which have limited cancer survivors’ ability to adhere to dietary and exercise recommendations. Moreover, the trained multidisciplinary team in SOAR is able to reinforce the effects of healthcare providers on participant lifestyle behaviors. Finally, SOAR participants were encouraged to journal, reflect, and practice throughout the duration of the program. These longitudinal activities likely contributed to the reinforcement of mindful eating concepts and the overall success of SOAR in increasing MEQ scores across multiple domains and across all BMI categories. 

Although this current study did not evaluate post-intervention BMI, SOAR increased the overall mindfulness of participants across all BMI categories. Compared to extant studies on teaching kitchens or mindfulness for cancer survivors, our study was unique in that it stratified participants into subgroups, with BMI >30 or <30, and measured MEQ scores across these two groups. Prior studies largely focused on survivors who were obese or overweight; both Thomas et al. 2019 and Sattler et al. focused on mindful eating in overweight or obese cancer survivors [32,41]. The observation that SOAR increased the overall mindfulness for participants in both BMI categories suggests that it can potentially serve as a means for both cancer survivors who wish to prevent weight gain, such as in those with lower BMI, and survivors who wish to lose weight. 

Compared to the few prior studies on teaching kitchens or mindfulness for cancer survivors, our participant demographics are similar. A total of 81% of our study participants reported their race as White, 13% reported their race as Black, and 7% reported their ethnicity as Hispanic. Most reported having some college education (26%) or a college degree (65%). Miller et al. evaluated the efficacy of a teaching kitchen for breast cancer survivors, focusing on a multidisciplinary approach that included psychosocial groups but did not evaluate mindful eating; the study found increased knowledge and confidence in preparing plant-based meals after intervention [42]. Similar to the demographics in our study, the participant demographics in Miller et al. comprised 77% non-Hispanic White, 8% Black, 4% Hispanic or Latino, and 11% other; 73% reported having completed college or postgraduate education. In Thomas et al. 2019, 96% participants reported their race as White/Caucasian [41]. The participant demographics in Sattler et al. comprised 90% White/Caucasian [32]. Chung et al. 2016 represents to date the only study focusing on mindful eating in African American breast cancer survivors [31]. Together, ours and another few studies continue to demonstrate promise in the application of mindful eating programs for cancer survivors’ eating habits; however, additional studies are needed with broader demographics distributions.

Our study recruited from all eligible breast cancer survivors at Moffitt Cancer Center and applied a pre- and post-test design with the same participants who represent their own control. We recognize the limitations of this design. The advantages of a pre-test post-test survey design without a control group are the simplicity of the study and the ability to detect a change within the group(s) over time. The observed changes are useful for evaluating the effectiveness of the program, and furthermore, this approach allows for comparison with a bulk of previously published work in the literature using this design. However, a pre-test post-test survey design without a control group may provide weaker evidence for causality and generalizability. Nevertheless, the design may demonstrate good internal validity when measurements are consistent. For example, one limitation of this study design is confounding by effects such as the testing effect, in which the same participant taking a survey may be influenced by having seen the survey previously [43]. This is, however, buffered by the absence of the effect in which survey instrumentation changes, i.e., our study used the MEQ both pre- and post-intervention. Moreover, the results’ reliability was enhanced as SOAR evaluated multiple mindful eating domains. 

The previous literature has suggested that the effects of mindfulness programs may wane over time [44,45]. Our study does not evaluate the sustainability of the approach including the impact of mindful eating on healthy eating indices outside the sessions, nor the effects of mindful eating on BMI, inviting further study into these questions. Finally, the generalizability of the study is limited by certain demographic factors of the study group such as low enrollment of Hispanic ethnicity, higher education levels, and familiarity and competence with online learning. Another challenge is sample recruitment bias as the participants self-enrolled in the intervention.

## 5. Conclusions

The SOAR intervention provides one of the first blueprints for a platform to teach mindful eating in a teaching kitchen for breast cancer survivors. Our mindfulness intervention showed improvement in the MEQ subscales of awareness, disinhibition, emotional response, and external cues. We believe mindful eating could be a useful tool to empower breast cancer survivors to adhere to the dietary guidelines for cancer risk reduction. In turn, this can lead to healthful behaviors compatible with the recommendations for reducing the risk of mortality and chronic disease. Indeed, the practice of mindful eating taught in the teaching kitchen setting may result in behavior changes that lead to healthier food choices and improvement in diet quality. Elements for success likely include the program’s multidisciplinary approach, its accessibility, and the level of participant interactivity. Moreover, further research is needed to examine whether mindful eating ultimately results in sustaining healthy eating behaviors and food choices. The results are promising that with guidance and mindfulness practice in a teaching kitchen, breast cancer survivors can develop the skills needed to modify behaviors for improved health outcomes. 

## Figures and Tables

**Table 1:** Participant demographics. Demographics of SOAR participants. gte30 indicates BMI ≥ 30 and lt30 indicates BMI < 30. “Missing” indicates where participants did not provide a response for that item.

Demographic Variable		N Participants Indicating Response	Total N
Cohort:	2020F	17 (16.7%)	102
	2021F	15 (14.7%)	
	2021S	12 (11.8%)	
	2022F	18 (17.6%)	
	2022S	15 (14.7%)	
	2023S	(24.5%)	
BMI Group:	gte30	49 (48.0%)	102
	lt30	(50.0%)	
	“Missing”	(1.96%)	
Pregnant:	Yes	0 (0.00%)	102
	No	99 (97.1%)	
	“Missing”	3 (2.94%)	
Gender:	Female	100 (98.0%)	102
	Other	2 (1.96%)	
	“Missing”	0 (0.00%)	
Age:	18–34 years	0 (0.00%)	102
	35–54 years	29 (28.4%)	
	55 or older	69 (67.6%)	
	“Missing”	4 (3.92%)	
Hispanic:	Yes	7 (6.86%)	102
	No	92 (90.2%)	
	“Missing”	3 (2.94%)	
Race:	Black	13 (12.7%)	102
	Other	4 (3.92%)	
	White	83 (81.4%)	
	“Missing”	2 (1.96%)	
Education:	College degree	66 (64.7%)	102
	HS degree	7 (6.86%)	
	Some college	26 (25.5%)	
	“Missing”	3 (2.94%)	

**Table 2:** Pre- and post-SOAR summary scores by mindful eating domains. Pre- and post-SOAR MEQ scores in each domain. *p*-values were calculated via Wilcoxon tests.

Scale	Pre-SOAR Mean (Sd)	Post-SOAR Mean (Sd)	Mean Change (Sd)	*p*-Value
Summary Score	2.84 (0.38)	2.96 (0.39)	0.12 (0.30)	0.0003
Awareness	2.58 (0.59)	2.73 (0.61)	0.15 (0.49)	0.0023
Distraction	3 (0.56)	3.01 (0.52)	0.01 (0.54)	0.6718
Disinhibition	3.06 (0.63)	3.18 (0.59)	0.12 (0.4)	0.0040
Emotional Cues	2.99 (0.77)	3.16 (0.67)	0.17 (0.54)	0.0034
External	2.56 (0.59)	2.7 (0.58)	0.15 (0.50)	0.0022

## Data Availability

The data presented in this study are available on request from the corresponding author.

## Supplementary Materials

The following supporting information can be downloaded at: https://www.mdpi.com/article/10.3390/nu15194205/s1, Table S1: Topics, Objectives, Activities and Recipes for Each SOAR Session.
