# Supplementary material for: Survivors Overcoming and Achieving Resiliency (SOAR): Mindful Eating Practice for Breast Cancer Survivors in a Virtual Teaching Kitchen
Source: Nutrients. 2023 Sep 29;15(19):4205. doi: 10.3390/nu15194205 (PMC10574766; doi:10.3390/nu15194205)
Supplement: Supplementary file 1 [file nutrients-15-04205-s001.zip › nutrients-2602486-supplementary.pdf]

SUPPLEMENTAL MATERIAL

Table S1: Topics, Objectives, Activities and Recipes for Each SOAR Session.

Session	Topic	Objective	Expert / Session Leader	Activity	Plant-based Recipes
1	Emotional Health & Well-being	Identify coping strategies essential to managing the stress, anxiety and depression common after a cancer diagnosis.	Licensed Clinical Social Worker	Journal and reflect on where you are in your cancer journey. Discuss coping strategies and when it's time to ask for help	Creamy cilantro lime dressing
2	Nutrition for Survivors	Learn the science behind nutrition and cancer survivorship.	Registered Dietitian	Separate on-line fact from fiction to maintain and improve health and survival. Journal and reflect on 1-2 dietary changes to move towards the AICR goals	Colorful lentil soup
3	Virtual Teaching Kitchen #1	Overcome barriers to consuming fish	Certified Culinary Medicine Specialist	Practice cooking skills and mindful eating while gaining knowledge of the health benefits of seafood	Sheet pan Alaska halibut
4	Exercise for Wellness	Discover the mental and physical benefits of physical activity for cancer survivors and its role in cancer risk reduction	Occupational and Physical Therapist	Directed practice in exercise activities using resistance bands adapted for your level of performance	Strawberry chia seed smoothie
5	Meditation & Mindful Eating	Use mindfulness to better understand and improve eating behavior.	Licensed Clinical Social Worker	Engage in a directed beginner's meditation and mindful eating fruit bowl exercise	Pomegranate salsa
6	Mindful Movement	Experience the benefits of mindful movement and yoga as a mind-body exercise	Certified Yoga Therapist	Directed practice linking gentle movements with the breath followed by reflection	Mango lassi overnight oats

7	Virtual Teaching Kitchen #2	Learn how plant-based eating benefits your health and the planet	Certified Culinary Medicine Specialist	Practice cooking skills and mindful eating while gaining knowledge of the health benefits of plant proteins	Spinach and Goat cheese stuffed portobellos
8	Medical Management	Learn what to expect at post-treatment check-ups and screenings	Medical Doctor	Identify strategies for effective communication with your healthcare team regarding managing long-term side effects	Strawberry aqua frescas
9	Moving forward with Arts in Medicine	Learn how to combine mindfulness with the arts as a tool for healing, self-awareness and self-expression	Artist in Residence	Reflect and write a healthy intention on the Origami paper and fold each peace calmly and mindfully with focused meditation	Citrus Quinoa Avocado Salad
